# Effect of Seat Angle when Sleeping in a Car on Quality of Sleep and Its Impact on Calculation Performance the Following Day

**DOI:** 10.3390/ijerph191912270

**Published:** 2022-09-27

**Authors:** Hitomi Ogata, Tomohiro Nishikawa, Momoko Kayaba, Miki Kaneko, Keiko Ogawa, Ken Kiyono

**Affiliations:** 1 Graduate School of Humanities and Social Sciences, Hiroshima University, Hiroshima 739-8521, Japan; 2 Department of Somnology, Tokyo Medical University, Tokyo 160-0023, Japan; 3 Graduate School of Engineering Science, Osaka University, Osaka 565-8531, Japan

**Keywords:** sleeping in a car, sleep architecture, seat angle, subjective sleep quality, calculation performance

## Abstract

The number of occasions to stay in a car overnight is increasing during disasters; however, the effects on sleep and the impact on daytime functioning are not well understood. We investigated the effect of seat angle when sleeping in a car and its impact on calculation performance the following day. Fifteen healthy males participated in three trials (sleeping in a car with the front seat angled at 45° and 60° in a laboratory and sleeping at home); sleep and calculation performance the following day were compared. Increased wake after sleep onset and decreased slow-wave sleep were observed in the 60° trial, that is, near-vertical, compared with the others. Subjective sleep quality and calculation performance in the 45° and 60° trials were poorer than those in the home trial. The effect of seat angle on sleep was confirmed objectively, but not subjectively, suggesting that a large seat angle might cause sleep impairment.

## 1. Introduction

The Red Cross reported that the number of natural disasters due to extreme weather and climate change, such as heat waves and floods, has risen by approximately 35% from the 1990s to the present [1]. Southeast Asia experiences the highest number of natural disasters in the world [2]. When natural disasters occur, many people are forced to evacuate their homes to public evacuation shelters and/or facilities. They are forced to sleep directly on the floor without sufficient privacy and air conditioning in public evacuation shelters such as school gymnasiums and community centers. In April 2016, the Kumamoto Earthquakes [3,4] (Japan Meteorological Agency) occurred, including a magnitude 6.2 foreshock earthquake on April 14, a magnitude 7.0 main shock on April 15, and subsequent numerous aftershocks. During the Kumamoto Earthquakes, the number of aftershocks that occurred at night was very high. Therefore, many victims were afraid to return to their homes and chose to evacuate; many survivors were forced to stay in their vehicles [5]. Indeed, it was reported that 74.5% of evacuees stayed in a car during the Kumamoto earthquake [6]. Moreover, during the coronavirus disease 2019 (COVID-19) pandemic, more people have chosen to stay in cars when they take refuge. However, few studies have examined overnight stays in cars.

Several studies have suggested that a more upright postural orientation inhibits sleepiness [7,8,9,10,11,12]. Aeschbach et al. [8] reported that subjects who slept in reclining chairs (rather than lying flat in bed) experienced reduced sleep efficiency, less rapid eye movement (REM) sleep, and increased light sleep (i.e., non-rapid eye movement, NREM 1). Similar results were reported by Nicholson and Stone [7]: adequate sleep may be obtained in seats as long as the back angle with the vertical approaches 40°. Four studies were conducted with participant napping or nocturnally sleeping using a car front seat. Horne et al. reported that naps enhanced arousal levels, and suppressed both subjective sleepiness and incidents, during a driving simulator task. According to a study conducted by Hayashi et al., short-term naps in car seats are more effective than no napping, and the closer the backrest angle is to the horizontal, the stronger the effect (130° vs. 150°) [10]. In our previous study, which compared sleep between a car front seat angled 45° and at home in nocturnal sleep, increased wake after sleep onset (WASO) and the number of stage shifts were confirmed [11]. Roach et al. [12] also reported a significant effect of back angle (20° (upright) vs. 40° (reclined) vs. 90° (flat)) on the latency to REM sleep, arousal, and the number of stage shifts. Thus, few studies have evaluated the effect of the seat angle on nocturnal sleep while sleeping in a car.

As mentioned above, our previous study showed that staying in a car overnight increased WASO and stage shifts [11]; however, the extent to which this impairment of sleep affected calculation performance the following day was unclear. In previous studies, sleep restriction [13,14] and sleep fragmentation [15] have been shown to decrease neurocognitive performance. Thus, sleep fragmentation caused by staying in a car overnight might cause deterioration in daytime performance.

Therefore, the purpose of this study was to objectively and subjectively investigate the effect of the sleep environment, that is, home and different front seat angles during overnight stays in a car on sleep quality and its impact on calculation performance the following day.

## 2. Materials and Methods

### 2.1. Participants

Fifteen young Japanese men (ages: 20.7 ± 0.6 years; Body Mass Index (BMI): 24.2 ± 4.4 kg/m^2^; Pittsburgh Sleep Quality Index (PSQI) score [16]: 4.8 ± 2.1; and Morningness-Eveningness Questionnaire (MEQ) score [17]: 46.4 ± 5.3) participated in the study. Exclusion criteria included food allergies, smoking, chronic diseases, back pain, shift workers, planned long-distance plane travel during the study period, self-reported sleep problems, and regular use of medications. The study was conducted in accordance with the guidelines of the Declaration of Helsinki and the ethics committee of Hiroshima University (approval number: 30-46-1) and was registered with the Clinical Trials UMIN (ID numbers: UMIN000046127, 20 November 2021). All participants provided written informed consent before the commencement of the study.

### 2.2. Study Design

**Figure 1:**
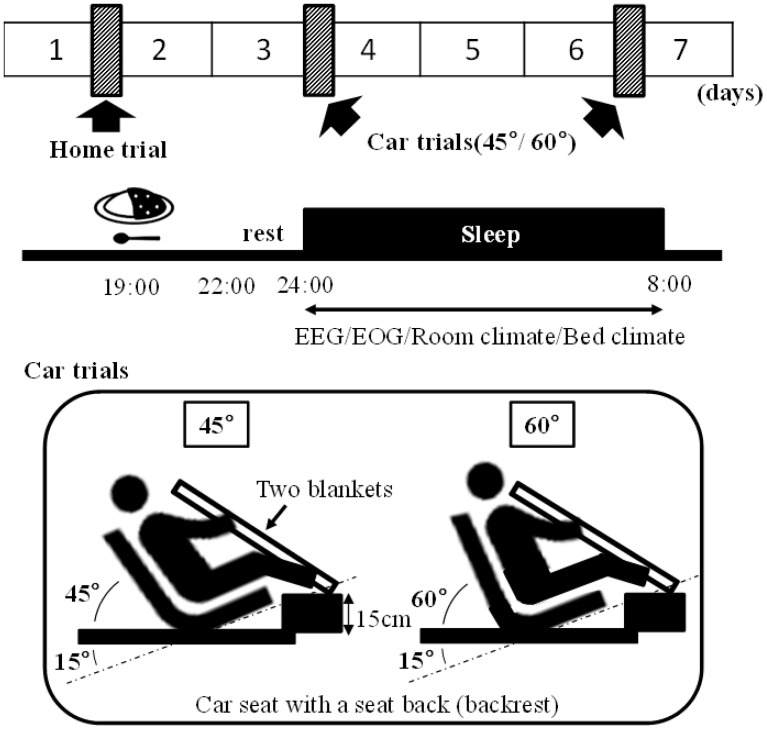
Study protocol: schematic overview of the study protocol (**top**); time schedule for sleep interventions for participants who always go to sleep at 00:00 (**middle**); and pattern diagram for each trial (**bottom**). All participants ate the same meals; dinner was curry and rice. On the day of the experiment, participants were restricted from caffeinated drinks, alcohol consumption, strenuous exercise, and naps, and had restrictions on television and smartphone use from dinner to the end of the experiment the next day.

The experiment was conducted from December 2019 to March 2020 in Hiroshima Prefecture, which is located in inland Japan. Before conducting this study, the participants practiced sleeping using a portable sleep device (see Section 2.3.2) at home to become familiar with the device. For each participant, the home trial (sleep at home) and car trials (sleep in the car front seat angled 45° and 60° placed in a laboratory) were conducted, giving a total of three trials. To prevent order effects, the order of car trials was alternately allocated. The washout periods were 2–8 days between the home trial and the first car trial, and 2–8 days between the car trials to avoid carry-over and seasonal effects. All three trials were performed within 2 weeks (Figure 1, top). Figure 1 (middle) shows the schedule for the day of the experiment. The participants ingested regular dinner 5 h before going to bed and went to bed at their usual bedtime (00:00 to 01:00) and slept for 8 h. On the day of the experiment, the participants were restricted from caffeinated drinks, strenuous exercise, and naps, and had restricted television and smartphone use from dinner until the end of the experiment the next day. Various measurements (see Section 2.3) were performed before going to bed, during bedtime, and after waking. In the car trials, two conditions were used: the backrest angle was 45° or 60° from the floor (60° or 75° from the seat surface): (1) 45°, which means that two people can stay on the back seat, and (2) 60°, which means that one person can stay on the back seat (Figure 1, bottom). The day before the sleep measurement, the participants were orally instructed to sleep at the prescribed time. In the car trials, two disaster rescue blankets (100% polyester, size 140 × 200 cm) were used as comforters, while in the home trial, bedding that the participants normally used at home was used. The participants took off their shoes and went to bed with their feet maintained 15 cm above the floor. The car trials were performed in a laboratory setting at 20 °C using heaters that were turned off during sleep. The participants wore long-sleeved tops and long pants; all three trials were conducted with the participants wearing their usual pajamas.

### 2.3. Measurements

#### 2.3.1. Room and Bedclothes Climate

As it was expected that the thermal environment would differ between the home and intervention trials conducted in the laboratory, the ambient temperature and relative humidity of the participant’s bedroom and climate in the bed (near the chest and near the feet) were recorded every 2 min using a data logger (TR-72Ui, T&D Corp., Matsumoto, Japan). The average ambient temperature and relative humidity during sleep were calculated.

#### 2.3.2. Objective Sleep Assessment

Sleep was recorded using portable two-channel electroencephalogram (EEG) and electrooculogram (EOG) monitoring systems (ZA-9, Proassist, Ltd., Osaka, Japan, [11]). Sleep stages (WASO, REM, NREM 1, NREM 2, and slow-wave sleep (SWS)) were manually scored according to the standard criteria [18] by a Certified Polysomnographic Technologist of the Japanese Society of Sleep Research without knowledge of the interventions. Previous studies reported that this portable two-channel device showed strong agreement with polysomnography (PSG), with kappa values of 0.80 overall [19] and 0.73–0.86 for REM, wake, NREM 2, and SWS [20], while the agreement with PSG was relatively low (κ = 0.44 [20]; inter-scorer concordance rates were 60.1% for the two-channel system and 71.7% for PSG [19]) in NREM 1. Sleep efficiency was calculated as the total sleep time (TST) per time in bed × 100. NREM 1, NREM 2, SWS, REM, and WASO were calculated as percentages of the TST. An overall stage shift, defined as the number of sleep stage transitions in sleep between the wake, NREM 1, NREM 2, SWS, and REM phases, was derived for each participant and used as a measure of sleep continuity.

#### 2.3.3. Subjective Sleep Assessment

##### Subjective Sleep Assessment

The Stanford sleepiness scale (SSS) score [21] was used to evaluate subjective sleepiness. This scale uses a 7-point Likert scale to quantify a participant’s sleepiness at the time the questionnaire was completed, with higher scores indicating greater sleepiness. The participants completed the SSS to rate their feelings of tiredness immediately before they went to bed and immediately after waking up.

##### Subjective Sleep Quality and Sleep Satisfaction

The Oguri-Shirakawa-Azumi (OSA) sleep inventory MA version [22] is a scale that measures subjective sleep quality upon waking; it is comprised of five subscales and 16 items that are self-evaluated on a 4-point scale: sleepiness on rising (e.g., “I have the ability to concentrate”), initiation and maintenance of sleep (e.g., “I slept very well”), frequent dreaming (e.g., “I frequently had a dream”), refreshing (e.g., “fatigue still remains”), and sleep length (e.g., “I had a long sleep”). The scores were calculated according to the manufacturer’s instructions.

Subjective sleep quality and sleep satisfaction were evaluated using the original visual analog scale (VAS) score. As for sleep quality and sleep satisfaction, we asked the participants, ‘Did you sleep well last night?’ (0 = I slept very well; 7 = I could not sleep at all) and “Are you satisfied with last night’s sleep?” (0 = very satisfied; 7 = very dissatisfied). The participants responded using the VAS immediately after waking.

##### Comfort in Sleeping Environment

A sleeping comfort questionnaire [23] was used to evaluate comfort in the sleeping environment as follows: warmth (1, hot to 5, cold), hardness (1, hard to 5, soft), comfort (1, bad to 5, good), humidity (1, wet to 5, dry), and turning over (1, difficult to 5, easy). The participants answered the sleeping comfort questions immediately after waking.

#### 2.3.4. Calculation Performance

To assess daytime performance, the participants performed a calculation task just before going to bed and immediately after waking up. A total of 174 calculation sheets were created, with 29 single digits horizontally and 6 vertically. The task format was based on the Uchida–Kraepelin test (National Institute of Mental Technology, Japan [24]). The time limit was set at 90 s. Because a total of six tasks, before and after sleep (two times) × trial (three conditions), were used for each person, six types of calculation tasks were prepared, and they were counterbalanced between participants. The numbers of responses and correct answers were then calculated.

#### 2.3.5. Statistical Analysis

Data were presented as mean values and standard deviations. One-way analysis of variance (ANOVA) and multiple comparisons using the Bonferroni test were used to compare the indoor climate, bed climate, and objective sleep assessments among groups. SSS score and calculation performance were evaluated by each value between before sleep and after waking up using a paired *t*-test in each group. For the relationship between the OSA sleep inventory and sleeping comfort, and the relationship between the score differences (the post-sleep minus pre-sleep) in the calculation tasks and objective/subjective sleep assessments, a partial correlation analysis was performed with participants as the adjustment variable to adjust for individual differences. To account for any missing EEG-based sleep-stage characteristic data, we used multiple imputations with a chained equation approach available in the MICE package of the R software. Multiple imputations can be used to avert the bias caused by missing data. We replaced each missing value with a set of substituted plausible values by creating 100 complete filled-in datasets. Log-transformation was applied to the variables with skewed distributions. All statistical analyses were performed using R version 3.6.0 (R Foundation for Statistical Computing, Vienna, Austria. http://www.R-project.org/, accessed on 19 March 2021). Differences were considered significant when the error probability was <0.05.

## 3. Results

### 3.1. Thermal Environment during Experimental Day

The average outside temperature at the site at the time of the experiment was 5.9 °C (average lowest −0.8 °C), and the average rainfall was 86.3 mm [25]. The room temperature was higher in the 45° trial (17.6 ± 0.8 °C) and in the 60° trial (17.5 ± 0.7 °C) than in the home trial (12.8 ± 0.7 °C), while the relative humidity was lower in the 45° trial (35.2 ± 1.4%) and in the 60° trial (35.7 ± 1.2%) than in the home trial (54.2 ± 0.8%). In all trials, the temperature decreased almost monotonically, and the relative humidity increased almost monotonically. The temperature in the bedclothes near each participant’s chest was lower in the 45° (26.8 ± 1.2 °C) and 60° trials (26.0 ± 1.3 °C) than in the home trial (28.6 ± 1.8 °C). The relative humidity was also lower in the 45° (35.9 ± 1.6%) and 60° trials (34.9 ± 2.1%) than in the home trial (44.4 ± 2.4%). In all trials, the temperature was increased until approximately 1 h after the start of the trial. Subsequently, there was no sudden change during the home trial; however, the temperature decreased moderately during the car trials. The relative humidity decreased almost constantly during the home trial, whereas it changed moderately in the car trials.

### 3.2. Objective Sleep Assessment

Nine trials that could not be measured successfully (removal of the electrode:7, measurement interval less than 6 h:2) and one home trial where the participant may not have been able to achieve normal sleep (sleep onset of home trial:85.0 min, 45° trial:17.5 min, 60° trial:49.0 min) were supplemented using the multiple imputation method. The sleep architecture results for each trial are presented in Table 1. ANOVA showed significant main effects of the trials for sleep efficiency, WASO, NREM 1, and SWS. According to the post hoc test, there was no difference in NREM 1, whereas WASO was significantly higher in the 60° trial than in the home and 45° trials. The SWS was also significantly lower in the 60° trial than in the home and 45° trials. Sleep efficiency tended to decrease in the 60° trial compared to the home trial.

### 3.3. Subjective Sleep Assessment

#### 3.3.1. Subjective Sleepiness

The SSS scores before going to bed were 3.1 ± 0.6 in the home trial, 3.5 ± 1.4 in the 45° trial, and 3.3 ± 0.8 in the 60° trial, and there was no significant difference between trials. The scores after waking up were 2.9 ± 1.2, 4.3 ± 1.4, and 4.4 ± 1.5, respectively; according to the post hoc test, the scores after waking were worse in the 45° and 60° trials than in the home trials.

The difference in the SSS scores for each trial after waking up was significantly lower than that of before sleep in the intervention trials (*p* = 0.452 for the home trial, *p* < 0.05 for the 45° trial, and *p* < 0.05 for the 60° trial).

#### 3.3.2. Subjective Sleep Quality and Sleep Satisfaction

The results of the OSA Sleep Inventory MA version for each trial are presented in Table 2. Sleepiness on rising, initiation, maintenance of sleep, and refreshing differed between the trials. According to the post hoc test, the scores for “sleepiness on rising” and “refreshing” were worse in the 45° and 60° trials than in the home trial, and the score for “initiation and maintenance of sleep” was worse in the 60° trial than in the home trial.

The VAS scores for sleep quality were 1.9 ± 1.4, 3.6 ± 1.9, and 4.9 ± 1.8 in the home, 45°, and 60° trials, respectively. The VAS scores for sleep satisfaction were 2.0 ± 1.5, 4.0 ± 1.8, and 5.2 ± 1.9. According to the post hoc test, the scores for both items were worse in the 45° and 60° trials than in the home trials.

#### 3.3.3. Comfort in Sleeping Environment

The results of the sleep comfort questionnaire after each trial are shown in Table 3. Hardness, comfort, and turning over differed between the trials. According to the post hoc test, the scores for hardness, comfort, and turning over were worse in the 45° and 60° trials than in the home trials.

#### 3.3.4. Correlation between Variables of OSA Sleep Inventory and Those of Sleeping Comfort Questionnaire

The results of the partial correlation analysis of the OSA sleep inventory and sleep comfort questionnaire revealed that the strongest correlation for sleepiness on rising in the OSA sleep inventory was turning over in the sleep comfort questionnaire (*r* = 0.480, *p* = 0.001), that for the initiation and maintenance of sleep in the OSA sleep inventory, it was comfortable in the sleep comfort questionnaire (*r* = 0.507, *p* = 0.001), and that for refreshing in the OSA sleep inventory, it was comfortable in the sleep comfort questionnaire (*r* = 0.653, *p* < 0.001).

### 3.4. Calculation Performance

**Figure 2:**
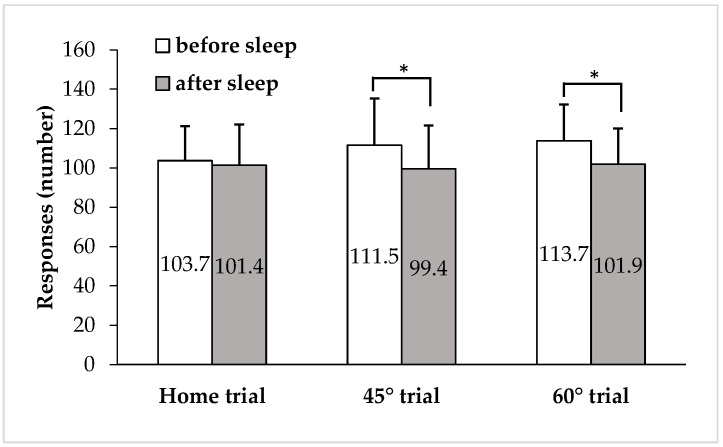
Number of responses in the calculation task (*n* = 15). Open squares show the results before sleep, and closed squares show the results after waking up. The number of responses was evaluated by the value between before and after sleep using a paired *t*-test in each group. * Significant difference before and after sleep (*p* < 0.05).

The results for the number of responses for each trial are shown in Figure 2. The differences in the numbers of responses between before sleep and after waking up were 2.3 ± 10.3 in the home trial, 12.1 ± 16.2 in the 45° trial, and 11.9 ± 13.9 in the 60° trial. The average number of incorrect answers for each trial before and after sleep was less than one.

The difference in the number of responses for each trial after waking up was significantly lower than that of before sleep in the intervention trials (*p* = 0.408 for the home trial, *p* < 0.05 for the 45° trial, and *p* < 0.01 for the 60° trial).

#### Correlation between the Result of Calculation Performance and Objective/Subjective Sleep Assessments

The results of the partial correlation analysis of the score differences (the post-sleep minus pre-sleep) in calculation tasks and objective/subjective sleep assessments revealed that the significant positive correlation for the sleep efficiency (*r* = 0.408, *p* = 0.017), sleepiness on rising in the OSA sleep inventory (*r* = 0.550, *p* = 0.001), and refreshing in the OSA sleep inventory (*r* = 0.497, *p* = 0.003) and the significant negative correlation for sleep latency (*r* = −0.378, *p* = 0.027), the sleep quality in the VAS score (*r* = −0.407, *p* = 0.017), and the sleep satisfaction in the VAS score (*r* = −0.430, *p* = 0.011).

## 4. Discussion

Because passenger cars are frequently chosen as emergency and evacuation places to stay during disasters and disaster recovery, it is necessary to evaluate the physical effects of overnight stays in cars to study disaster countermeasures. Therefore, this study aimed to reproduce the sleep environment with two types of front seat angles, that is, 45° and 60° from the floor, in healthy young adults, and to clarify the impact of seat angle on the quality of sleep and its impact on calculation performance.

We first confirmed whether the thermal environment affected the sleep architecture. There were significant differences in temperature and relative humidity between the home and intervention trials, similar to our previous study [11]; however, there was no significant difference among the trials subjectively, that is, the sleeping comfort questionnaire. The present study confirmed that intervention trials, that is, sleeping in a car seat angled at both 45° and 60°, disturbed objective and subjective sleep compared with the home trial, which was consistent with our previous study. Moreover, the calculation performance indicated by the number of responses after waking up worsened in that of before sleep in the intervention trials. The increase in WASO in the intervention trials was also consistent with a previous study where WASO increased, as measured by EEG and EOG monitoring systems, in a car seat trial compared with a home trial [11]. In another study, WASO increased as measured using an actigraph under conditions recreating the sleep environment of an evacuation shelter in winter [26]. In addition, the feet were maintained 15 cm above the floor during sleep in our study. Compared to our previous study [11]), we obtained better sleep quality, despite the same seat angle; for example, WASO was 7.4%, and stage shift was 81.1 times. It has been suggested that keeping the feet slightly higher may also improve sleep quality. Contrary to our expectations, the number of stage shifts, which indicates that sleep is fragmented [27], did not significantly increase in the intervention trials. This was because only participants who could sleep well according to the PSQI participated in the study. Cicolin et al. reported that the average number of stage shifts in healthy young people was 73.5 times (total sleep time, 363.7 min) [28], while our results were very low, despite the car trials. In addition, the results of the partial correlation analysis between the OSA sleep invention and sleeping comfort question suggest that “turning over” or “sleeping comfort” may be significantly related to the subjective quality of sleep. Additionally, the results showed a partial correlation between the results of the calculation performance and objective/subjective sleep assessments, sleep latency and sleep efficiency in the objective sleep assessments and the VAS score and OSA sleep invention in the subjective sleep assessments.

Our study revealed that the subjective quality of sleep did not differ between the intervention trials, while the effect of different front seat angles during overnight stays on sleep quality was objective. There were no significant differences in the thermal environment between the intervention trials. Previous studies using car seats have shown that short-term naps lead to lower subjective drowsiness than non-napping, reduce the number of accidents during simulated driving, and improve task performance [9,10]. Moreover, a seat angle closer to the horizontal is more beneficial than a more perpendicular seat angle [10]. Consistent with previous studies [7,8,12], the difference in objective sleep between the intervention trials could be caused by the effect of differences in seat angles. These results may be explained by two main mechanisms. First, it is difficult to maintain the head in a comfortable position for sleep when sitting upright, which is likely exacerbated during REM sleep when the muscle tone is very low. Second, the upright posture increases sympathetic activity and decreases parasympathetic activity, resulting in a heightened level of physiological arousal; however, we did not assess autonomic activity, so this needs to be assessed in the future. While WASO and SWS differed between the 45° and 60° trials, there were no differences in the subjective sleep quality. A previous study reported that subjective and objective drowsiness deviated from each other in various cases [29]. Although the mechanism whereby the objective and subjective results do not match between the 45° and 60° trials was not determined in the present study design, this result may indicate that the participants were unaware of their sleep deprivation during the car trial. The lack of differences between the car trials regarding subjective sleep assessments indicates that there was a risk of falling asleep after selecting a backrest angle without realizing that would lead to a decrease in the amount of sleep. This indicates that an inappropriate seat angle would cause further sleep impairment while staying in a car overnight. Our study was a temporary intervention experiment; however, the accumulation of unconscious sleep problems may lead to mental and physical health issues. In future, it is also necessary to consider continuous effects based on actual evacuation situations. Moreover, insufficient sleep leads to sleep debt, which is an accumulated amount of sleep loss that has a harmful impact on carbohydrate metabolism, endocrine function, and the kidney [30,31].

Our study had some limitations. First, a portable two-channel EEG and EOG was used in the present study to measure sleep stages with objectivity at home, although its accuracy, which is judged in NREM 1, was limited compared to PSG. Second, the participants were verbally instructed to adhere to sleep controls the day before the experiment, but this was not confirmed using objective measures. Although the subjective questionnaire confirmed the psychological aspects of the participants’ conditions, the duration of sleep on the previous day may have affected the results. Thirdly, it cannot be determined whether the difference in the results is simply the backseat angle because the control trial was carried out at home. The effects of sleep comfort on the back seat are yet to be evaluated. Fourthly, the noise that imitates engine sound was not examined. Listening to music is a widely used tool to improve sleep [32], whereas sound levels are negatively correlated with sleep duration in patients in an intensive care unit [33]. Finally, the applicability of results to real-life situations is limited due to the selection of people with good sleep and health in the present experiments. Further studies targeting women and the elderly are also needed, and the chronic effects of sleep remain to be investigated.

## 5. Conclusions

To summarize, the sleep environments in a car (two backrest angles) were reproduced experimentally to compare sleep quality in cars and at home. As the results of the subjective sleep assessments and calculation performance were not significantly different between the car trials, although the results of the car trials were significantly worse than those of the home trials. According to the results of the objective sleep assessment, the WASO was significantly higher in the 60° trial than in the home and 45° trials. The SWS was significantly lower in the 60° trial than in the home and 45° trials. The effect of seat angle on sleep was confirmed objectively, but not subjectively, suggesting that a large seat angle might cause sleep impairment.

## Figures and Tables

**Table 1:** Results of sleep stages using the portable two-channel electroencephalogram monitoring system and supplemented using the multiple imputation method.

		Home Trial	45° Trial	60° Trial	*p*-Value
TST	(min)	437.5 ± 53.4	428.8 ± 54.3	405.8 ± 40.5	n.s
REM latency	(min)	16.2 ± 13.9	36.3 ± 58.5	30.6 ± 34.1	n.s
Sleep latency	(min)	87.9 ± 30.3	110.6 ± 59.0	128.2 ± 66.8	n.s
Sleep efficiency	(%)	95.0 ± 3.7	89.3 ± 11.3	85.1 ± 9.0 §	0.008
WASO	(%)	1.5 ± 1.1	3.2 ± 2.5	9.0 ± 8.3 † ⁑	0.001
REM	(%)	22.7 ± 4.2	23.4 ± 4.1	20.6 ± 5.4	n.s
NREM 1	(%)	2.4 ± 0.9	3.4 ± 2.0	4.7 ± 3.3	0.026
NREM 2	(%)	53.8 ± 7.0	52.4 ± 5.8	52.8 ± 7.6	n.s
SWS	(%)	19.6 ± 4.6	17.6 ± 5.9	12.9 ± 5.0 † ⁑	<0.001
Stage shift	(times)	41.1 ± 15.6	51.9 ± 24.8	60.6 ± 38.1	n.s

Values are means ± SDs; *n* = 15. TST, total sleep time; REM, rapid eye movement; WASO, wake after sleep onset; NREM, non-rapid eye movement; SWS, slow-wave sleep; percentage divided by TST. One-way ANOVA was used to evaluate the effect of trials on sleep assessments. As a post hoc test, multiple comparisons using Bonferroni correction were conducted. † vs. home trial *p* < 0.05, ⁑ vs. 45° trial *p* < 0.05, § vs. home trial *p* < 0.1.

**Table 2:** Results of OSA sleep inventory MA version.

	Home Trial	45° Trial	60° Trial	*p*-Value
Sleepiness on rising	18.5 ± 3.9	11.9 ± 5.0 *	11.0 ± 6.6 †	<0.001
Initiation and maintenance of sleep	15.1 ± 6.2	11.7 ± 5.8	8.9 ± 5.8 †	0.006
Frequency dreaming	19.0 ± 8.0	18.7 ± 8.9	19.0 ± 7.2	n.s
Refreshing	20.6 ± 4.5	11.8 ± 8.0 *	11.0 ± 6.9 †	<0.001
Sleep length	21.6 ± 7.7	17.9 ± 4.0	14.9 ± 6.7	0.048

Values are presented as the mean ± SD; *n* = 15. A high score indicated a good subjective feeling of sleep. One-way ANOVA was used to evaluate the effect of trials on OSA sleep inventory MA version. As a post hoc test, multiple comparisons using Bonferroni correction were conducted. * vs. home trial *p* < 0.05, † vs. home trial *p* < 0.05.

**Table 3:** Results of the sleeping comfort questionnaire.

	Home Trial	45° Trial	60° Trial	*p*-Value
Warmth feeling	3.2 ± 0.6	3.7 ± 0.7	3.7 ± 0.8	n.s
Hardness	3.3 ± 0.6	2.3 ± 0.8 *	2.1 ± 0.8 †	<0.001
Comfort	4.2 ± 0.8	2.7 ± 0.8 *	1.9 ± 0.8 †	<0.001
Humid feeling	3.1 ± 0.6	3.3 ± 0.7	3.3 ± 0.7	n.s
Turning over	3.4 ± 1.0	2.0 ± 0.8 *	1.5 ± 0.6 †	<0.001

Values are presented as the mean ± SD; *n* = 15. A high score indicates a poor subjective feeling of sleeping comfort. One-way ANOVA was used to evaluate the effect of trials on the sleeping comfort questionnaire. As a post hoc test, multiple comparisons using Bonferroni correction were conducted. * vs. home trial *p* < 0.05, † vs. home trial *p* < 0.05.

## Data Availability

Data can be obtained from the corresponding author upon a reasonable request.
